# A survey of oral health in a Sudanese population

**DOI:** 10.1186/1472-6831-12-5

**Published:** 2012-02-24

**Authors:** Nadia Khalifa, Patrick F Allen, Neamat H Abu-bakr, Manar E Abdel-Rahman, Khalda O Abdelghafar

**Affiliations:** 1 Prosthodontic Department, Faculty of Dentistry, Khartoum University, Khartoum, Sudan; 2 Department of Restorative Dentistry, Cork University Dental School & Hospital, Wilton, Cork, Ireland; 3 Conservation Department, Faculty of Dentistry, Khartoum University, Khartoum, Sudan; 4 School of Maths, Khartoum University, Khartoum, Sudan; 5 Department of Statistics, Federal Ministry of Health, Khartoum, Sudan

## Abstract

**Background:**

We aimed to assess the oral health status and risk factors for dental caries and periodontal disease among Sudanese adults resident in Khartoum State. To date, this information was not available to health policy planners in Sudan.

**Methods:**

A descriptive population-based survey of Sudanese adults aged ≥ 16 years was conducted. After stratified sampling, 1,888 adult patients from public dental hospitals and dental health centres scattered across Khartoum State, including different ethnic groups present in Sudan, were examined in 2009-10. Data were collected using patient interviews and clinical examinations. Dental status was recorded using the DMFT index, community periodontal index (CPI), and a validated tooth wear index.

**Results:**

Caries prevalence was high, with 87.7% of teeth examined having untreated decay. Periodontal disease increased in extent and severity with age. For 25.8% of adults, tooth wear was mild; 8.7% had moderate and 1% severe toothwear. Multivariate analysis revealed that decay was less prevalent in older age groups but more prevalent in southern tribes and frequent problem based attenders; western tribes and people with dry mouths who presented with less than18 sound, untreated natural teeth (SUNT). Older age groups were more likely to present with tooth wear; increasing age and gender were associated with having periodontal pocketing ≥ 4 mm.

**Conclusions:**

The prevalence of untreated caries and periodontal disease was high in this population. There appear to be some barriers to restorative dental care, with frequent use of dental extractions to treat caries and limited use of restorative dentistry. Implementation of population-based strategies tailored to the circumstances of Sudanese population is important to improve oral health status in Sudan.

## Background

According to the World Health Organisation (WHO), "oral health means being free of diseases and disorders that affect the mouth and oral cavity" [1]. Several factors including social [2], behavioural [3], and medical [4] seem to play a role in oral disease progression. Descriptive population health surveys provide a basis for estimation of the oral health status of a population and its future needs for oral health care.

Dental caries experience is commonly recorded using the decayed, missing, and filled teeth (DMFT) index [5]. Mean DMFT scores are used to give an estimate of caries prevalence and its treatment (either by tooth extraction of restorations). Periodontal status in population studies is recorded using the Community Periodontal Index (CPI) [5]. The main outcome measures of CPI are presence of gingival bleeding on gentle probing, dental calculus, and probing periodontal pocket depth (PPD): 4-5 or ≥ 6 mm.

Partial mouth recordings have been utilized to record level of tooth wear [6,7] and partial recording of 12 anterior teeth was found appropriate to measure tooth wear [8].

There is a high prevalence of oral disease globally and the consequences of oral disease pose substantial public health problems including pain, impairment of function and, reduced quality of life [9]. In the US population, oral conditions caused more days of work loss than stroke, and in younger adults, as much work loss as all neoplasia combined [10].

Decision makers and health planners need information about risk factors for oral disease to help identify individuals who are at risk of developing oral disease and to target population level interventions. This includes the need to collect data on social and medical status, health behaviours and demographic data in addition to clinical data. Only a few studies exist considering adult oral health in sub-Saharan Africa [11-16]. Findings of these studies suggest that the prevalence of dental disease is generally low in African populations, and that limited access to dental services leads to retention of carious teeth.

There is a relative lack of data pertaining to adults in the developing world in general and in Sudan in particular. Most of the studies focused on school children or used small samples.

The aim of this study was to assess the oral health status and associated risk indicators for oral disease in Sudanese adults attending outpatient clinics in Khartoum State.

## Methods

### Study design

**Figure 1:**
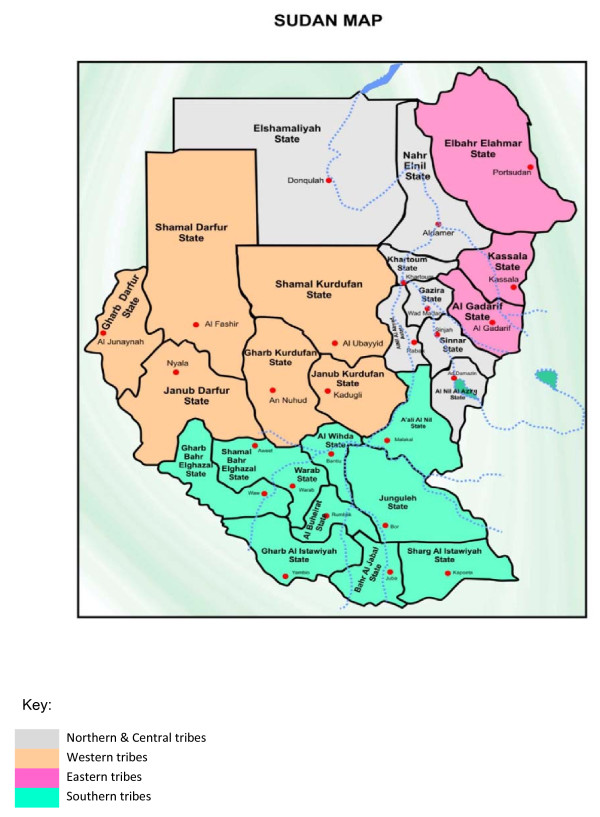
**Map of Sudan**.

This cross-sectional oral health survey was part of a study designed to assess the functional and psychosocial impact of dental disease and was carried out between August 2009 and March 2010. The study participants were recruited from among those attending outpatient dental hospitals and health centers distributed among the seven provinces (Um Durman, Khartoum Bahri, Khartoum, Jabal Aulia, Sharg En Nile, Karary, and Um badda) of Khartoum State. Sudan was the largest country in Africa before South Sudan became an independent country on 9 July 2011. It was divided into 25 states, with Khartoum State the capital being the most densely populated state (Figure 1). The study population comprised 1,888 patients. The sample size was calculated using the formula for proportion estimates considering a tooth loss prevalence of 67% according to previous Sudanese studies [17,18] and precision of 3; the design effect was set as 2. The sampling frame for the study was the public dental service in Sudan. There are 3 levels (federal, state, and locality) of health care systems in Khartoum. All the dental outpatient clinics of these facilities were included in the sampling frame. The sample size of each outpatient clinic was obtained by the following equation: *n_h _*= (*N_h_*/*N*)*n; where *n_h _*was the sample size of each outpatient clinic *h, N_h _*the population size (no. outpatients/3 months of the specific hospital/dental health center [DHC]) for stratum *h, N *the total population size (total no. outpatients/3 months of all hospitals and DHC), and *n *total sample size (1,888) (Table 1). Patients were selected consecutively until the required number of patients from the different hospitals and DHCs were obtained. Written consent was obtained from all patients. The study protocol was approved by the National Ethical Clearance Committee of the Federal Ministry of Health in Khartoum, Sudan.

**Table 1:** Proportional sample size calculation of hospital/dental center

*H*	*N_h_*	*n_h_*
Khartoum Teaching Dental Hospital	21,000	343

Umdurman Dental Hospital	23,275	382

Bahrey Dental Hospital	13,046	211

Police Dental Hospital	24,500	400

Military Dental Hospital	19,784	323

Dental Health Centers	14,025	229

	115,632 = **N**	1,888 = **n**

### Data collection

Socio-demographic variables included age, gender, ethnic group, and socioeconomic status (occupation, total monthly income, education).

Behavioural variables included frequency and reason for dental visits, if applicable number of teeth removed at final visit, tobacco use and way of consumption, as well as frequency and method used for dental hygiene.

Because of the developing interest in the relationship between oral and general health, it was considered necessary to try to establish some of the main medical characteristics of the population sample through questions such as use of medication, previous surgery and details of diagnosed medical conditions. Participants were also asked "How often does your mouth feel dry?" with response options "always", "frequently", "occasionally", or "never". At the analysis stage, those who had responded "always" or "frequently" were designated as xerostomic.

Data were collected using a questionnaire administered in interviews by researchers not involved in the treatment of the patients. The interviews took 15-20 minutes prior to the clinical examinations.

Clinical examinations were undertaken by five calibrated dentists, including the lead author. Following a period of training in clinical examination procedures and calibration, inter-examiner reliability was checked using intra-class correlation coefficients (ICC). Inter-examiner reliability was assessed in 20 patients at the beginning of the survey, and during the survey. Field checks were also carried out during the survey by the main investigator who also acted as gold standard.

Inter-examiner reliability was assessed by intra-class correlation coefficient (ICC) on clinical measures of CPI, DMFT, and tooth wear at 2-3 weeks apart. ICC for CPI, DMFT, and tooth wear the before start of survey was 0.67 (95%CI, 0.56-0.83), 0.96 (95%CI, 0.92-0.97), and 0.55 (95% CI, 0.41-0.75), respectively, and during the survey was 0.61 (95% CI, 0.55-0.68), 0.85 (95% CI, 0.81-0.87), and 0.59 (95%CI, 0.49-0.62), respectively. Thus, according to Fleiss [19], ICC for CPI ranged from fair to good, for DMFT excellent, and for tooth wear fair to good.

Once a satisfactory level of examiner reliability was established, clinical examinations were undertaken using WHO criteria for population oral health surveys.

DMFT was used to obtain estimates of how much the dentition was affected by dental caries. The clinical examination included a full mouth recording for 32 teeth [5]. Decay was recorded if a carious cavity was visually present and a CPI probe was used to confirm visual evidence of caries. A tooth was recorded as missing due to caries if there was a history of extraction because of the presence of a cavity prior to extraction. Periodontal health was assessed by CPI [5]. The three indicators used for this assessment were gingival bleeding, calculus, and periodontal pockets. A specially designed lightweight WHO CPI probe was used to record clinical data by sextant, and coded as 0 (no disease), 1 (gingival bleeding detected), 2 (calculus detected), 3 (pockets ≤ 5.5 mm) or 4 (pockets of ≥ 6 mm)

The 12 upper and lower anterior teeth were examined for toothwear. The index used was that used in the survey of Oral Health in Irish Adults 2000-02 [20] and the Adult Dental Health Survey in the United Kingdom in 1998 [7]. It was a descriptive index using partial recording of the labial, incisal, and palatal surfaces of the upper six permanent anterior teeth. On the upper incisal surfaces, wear typical of erosion was scored if present. The condition of the most worn surface of the lower six permanent anterior teeth was recorded. Wear was recorded when it had progressed through tooth enamel into the dentine because considerable inter-examiner variability has been reported when trying to record wear confined to tooth enamel.

Tooth wear was classified as "mild" (tooth wear just exposing the dentine), "moderate" (tooth wear exposing the dentine for more than one third of the individual surface), or "severe" (complete loss of tooth enamel, with the pulp or secondary dentine exposed).

All data were recorded on standardized proformae and entered into a spreadsheet for analysis. Random checking was undertaken to verify the accuracy of data entry.

### Data analyses

Analyses were performed using the statistical software package STATA Release 9 (Stata Statistical Software 2005; StataCorp LP, College Station, TX, USA). Summary data were reported using frequency distributions. The categorical-dependent or outcome variables were reduced to binary variables such as: < 18 and ≥ 18 sound untreated natural teeth (SUNT); zero decayed teeth (DT) and ≥ 1 DT; no tooth wear and tooth wear; healthy periodontal tissues and those with periodontal pockets ≥ 4 mm. Bivariate analysis of these data was undertaken using Pearson's chi square tests. The independent factors used in these analyses included sociodemographic such as sex, age group, ethnic group, occupation, monthly household income, and education level achieved; behaviours such as frequency of dental visits, tobacco use, and frequency and type of dental hygiene and,; medical, such as how often mouth feels dry, history of surgery, and, current medical status. Multivariate logistic regression modeling was used to ensure allowance for potential confounding variables.

## Results

### Sociodemographic, behavioral, and medical characteristics

The number of adults examined was 1,888 split into seven age groups "see Additional file 1". According to their ethnic group, most probands (57%) came from northern and central tribes followed by western tribes (33.7%). For nearly three quarters of subjects, the head of household had an income of < 250 SDG (< 75 Euro monthly); 25.8% never went to school or only went to khawla, a type of kindergarten, with 20% completing only primary school and 60% having semiskilled or unskilled jobs.

Over sixty percent of subjects went to the dentist less frequently than every 2 years, 16.7% went more frequently than every 2 years, and 22.7% never went, indicating poor attendance. Only 9% went for regular checkups whereas > 91% of patients only went to the dentist when they were in pain.

In terms of treatment received during the most recent dental visit, more than 55% of people had a single tooth extraction as their only treatment. When asked about the reason for extraction, nearly 80% stated that this was the advice given by the dentist.

Tobacco use was prevalent in approximately 17%. of the sample. Besides cigarette smoking (62%), the use of smokeless tobacco, locally known as toombak, was reported in 51% of the sample.

In terms of dental hygiene behaviours only, 53% reported that they brushed their teeth twice daily. Additional methods of oral hygiene such as use of a mouth rinse (11%) and inter-dental cleaning (3%) were rarely used.

Of the people who reported medical problems (17%), 27% had hypertension and 17% diabetes mellitus. This was also reflected in similar pattern by people on medication (14%) among whom 21% were on antihypertensive and 14.6% on antidiabetic medications. The feeling of dry mouth was assessed using a validated xerostomia index [20]. In response to the question "How often does your mouth feel dry?" (response options: always, frequently, occasionally, or never), those who had responded with "always" or "frequently" were designated as xerostomic. Nearly one fifth (19.3%) of participants reported that their mouth felt dry occasionally or more frequently, with 3.5% to the point of being xerostomic.

### Frequencies of clinical findings: CPI, DMFT, tooth wear

**Figure 2:**
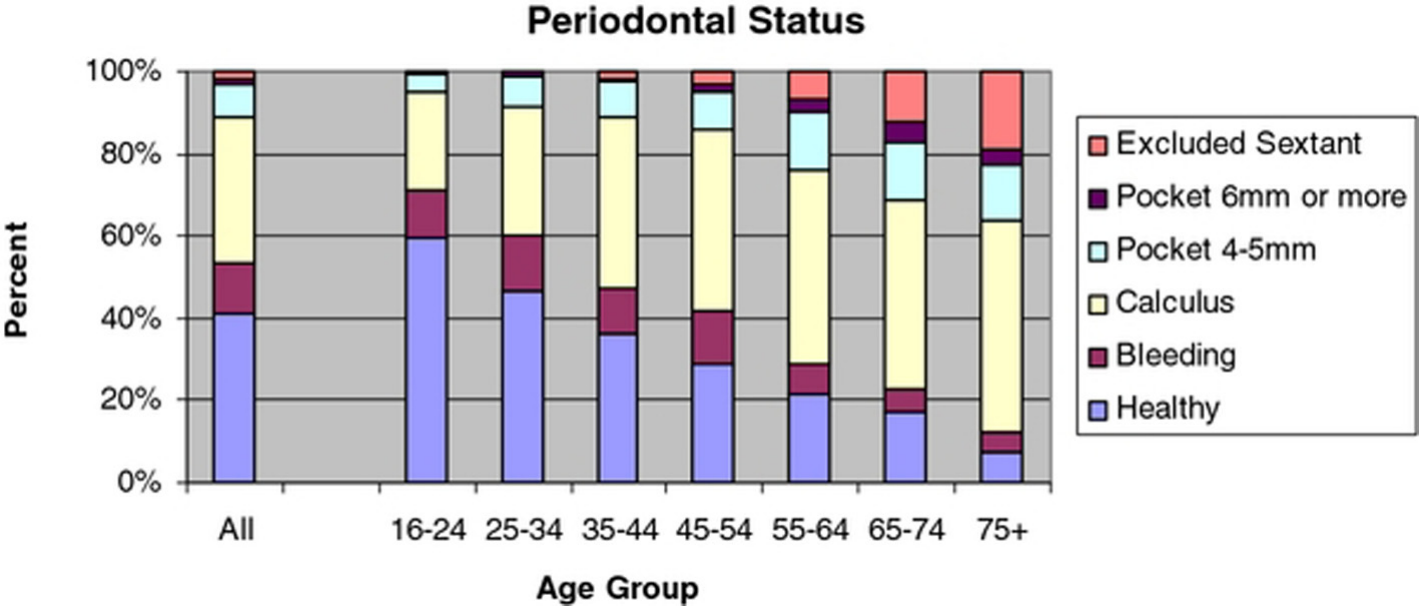
**Distribution of adults according to periodontal conditions**.

In the 35-44 age group 36.1% had healthy periodontal tissues, 10.9% bleeding, 42.0% calculus, 8.5% 4-5-mm periodontal pocketing, 0.7% periodontal pocketing of ≥ 6 mm, and 1.8% excluded sextants (Figure 2).

The mean DMFT for age group 35-44 years was 8.7 (SD, 5.9) (Table 2). The overall mean number of missing teeth was 3.6 (SD, 4.9) and in the age group 35-44 years was 4.2 (SD, 4.1). Surprisingly, the mean number of teeth was high 28.4 (SD, 4.9); however, percent DT was 87%. Even though teeth existed, they were badly decayed due to lack of treatment. This was substantiated by the finding that the filled component of DMFT was 0.2%. The prevalence of root caries in adults aged ≥ 16 years on exposed teeth was 23.6%. Root caries was more prevalent with increasing age.

**Table 2:** Mean DMFT according to age group

*Age group*	*N*	*Mean* *DMFT*	*Std. Deviation*	*Std. Error of mean*	*Mean* *D(SD)*	*Mean* *M(SD)*	*Mean* *F(SD)*
16-24	413	4.2	3.4	0.2	2.9(2.6)	1.2(1.9)	0.1(0.6)

25-34	616	5.5	4.1	0.2	3.3(2.9)	1.9(2.5)	0.3(1.0)

**35-44**	**368**	**8.7**	**5.9**	**0.3**	**4.1(3.4)**	**4.2(4.1)**	**0.3(0.9)**

45-54	253	9.8	6.8	0.4	4(4)	5.5(5.4)	0.2(0.7)

55-64	133	12.2	8.3	0.7	3.9(4)	8(6.5)	0.3(0.8)

65-74	77	14.4	8.0	0.9	3(2.9)	11.3(7.9)	0.2(0.6)

75+	22	15	10.8	2.3	3.3(3.9)	11.8(10)	0(0)

Total	1,882	7.4	6.2	0.1	3.5(3.2)	3.6(4.9)	0.2(0.8)

More than one third (35.5%) of adults had some degree of wear of their anterior teeth that involved at least some dentine. In 25.8% of adults the wear was mild; 8.7% had moderate and 1% severe wear. Mild tooth wear decreased with age whereas moderate and severe increased with age.

### Multivariate logistic regression analysis

Independent factors found significant by Pearson's chi square analysis were all entered in one step, into four separate multivariate logistic regression models, investigating the likelihood of having decay, ≥ 18 SUNT, tooth wear, and periodontal pockets ≥ 4 mm. Details of variables with increased/decreased odds of predicting oral disease are presented "see Additional file 1", with R^2 ^values showing how much variation can be explained by each model. The multivariate logistic regression analysis revealed that presence of decay seemed less prevalent in older than younger age groups. Those who were educated, went for checkups, and cleaned between teeth had a decreased likelihood of having dental decay. Western tribes (OR, 1.83) as well as those with occasional dry mouth (OR, 2.18) were more likely to present with < 18 SUNT.

As expected, older age groups presented more tooth wear than younger age groups. In terms of periodontal disease, increasing age and being a male were characteristics associated with periodontal pocketing ≥ 4 mm.

## Discussion

This study is the first large population based study of adult oral health in Sudan. There were some limitations that should be considered when interpreting the data presented in this study. Given the available infrastructure, it is not possible to conduct a randomly selected sample representative of the entire Sudanese population. Whereas it is possible to obtain some information on oral health issues from patients attending outpatient facilities of hospitals and dental health centres of Khartoum State, the findings may not be representative of the whole of Sudan. The sample was biased in that visits by the individuals attending these clinics were problem based. However, the sampling strategy employed ensures that the sample recruited from the country's most populated state is broadly representative of Sudan. Given the limited infrastructure for oral health services delivery in Khartoum, the prevalence rates of conditions reported in this paper are unlikely to be overestimated. Many people in Sudan do not receive regular dental care and have acute problems when seen by a dentist. In Sudan the dentist-to-patient ratio is 1:33,000 compared with approximately 1:2,000 in most industrialized countries [21]. Relative to the size of the Sudanese population, there are very few dentists and this restricts access to regular dental care. Other factors which influence dental attendance in Sudan include the lack of public funding for oral healthcare and dental insurance schemes to ameliorate the cost of care. In that sense, the dental attendance experience of this sample is not untypical of the wider Sudanese context.

One of the most striking findings from the study is the apparent lack of restorative or preventive dental care, as shown by the filled component F (0.2%) and treatment is limited to pain relief or emergency care by tooth extraction. Dentists' attitudes toward dental treatment were shown greatly to influence tooth extractions in this study. It would appear that there are barriers to the provision of restorative dental care which are multifactorial in origin and worthy of further investigation.

There were relatively few participants aged ≥ 65 years, but this reflects the age distribution of the Sudanese population according to the population Census of Khartoum State 2007 [22]. In Khartoum state, only 2% of the population are aged over 65 years. Also of interest is the observation that life expectancy at birth of Sudanese is 55-60 years according to United Nations [23]. People from older age groups might also have lower expectations or less money available for dental treatment.

The low level of literacy observed in Khartoum (which is probably amplified in other Sudanese states) as well as low income level, could have had profound effects on the level of oral health observed in this study. The impact of socioeconomic status on oral health has been documented in other studies [24,25].

In terms of accessing dental care, only 16.7% reported attending more frequently than every 2 years, which is much lower than reported elsewhere. For example, the Irish Oral Health Survey of adult oral health in 2000-02 reported that 44-57% visited the dentist more frequently than every 2 years.

Because nearly one fifth of subjects used tobacco in some form, the strong correlation between smoking habits, severity of periodontal disease, and tooth mortality, as established in various studies, should be considered [26,27]. Smokeless tobacco "Toombak" has been linked to oral health hazards such as cancer in a few studies [28,29].

The finding that just over half the population brushed their teeth twice daily is similar to the findings of the Irish National Oral Health Survey 2000-02 [20], whose authors commented that people who brushed at least twice daily had a greater number of teeth and lower DMFT and were more likely to have ≥ 18 SUNT. Very few participants used additional methods of oral hygiene such as floss or mouthwashes, and this might be due to lack of awareness or inability to purchase them.

The prevalence of xerostomia in Khartoum at 3.5% was much lower than in other studies, where approximately one fifth of older people [30], and 10% of subjects aged in their early 30s [31] reported the condition. This might be due to more limited exposure of the Sudanese population to medications which reduce saliva flow. Further investigations using objective besides subjective measures for xerostomia are needed to observe whether any associations between medications or other factors such as medical condition and xerostomia exist; this is beyond the scope of the present study.

In this study, it was decided to use CPI to give an indication of the periodontal status of subjects. There are, however, some shortcomings to CPI such as it does not distinguish between gingival inflammation and periodontal destruction because of its hierarchical scoring principle. Furthermore, the use of index teeth instead of a full mouth recording has been shown to increase underestimation of the prevalence of periodontal pockets [32]. However, the use of alternative indices would allow direct comparison with only a few other studies, which is the reason that we decided to use the CPI in this study. The results of CPI were very different to those of a study carried out in Sudan in by Ali in 1991 [33] who included 126 adolescents and 138 adults from areas inside as well as around cities of Khartoum and El Obeid. In that study of adults aged 35-44 years, only 3% had calculus, 71.3% had probing pocket depth between 4 and 5 mm, and 25.7% ≥ 6 mm. These results revealed a much higher prevalence and severity of periodontal disease than our study, which could be due to a combination of factors such as differences in sample design. The more severe periodontal disease could also have resulted from a decreased awareness towards oral health at the time when Ali's study was carried out almost 20 years ago [33]. Geographic region could have also played an important role because approximately half the population studied was from Obeid, which is outside Khartoum State. One could speculate that different methods of dental self-care might have been more common then, such as using the miswak (a teeth-cleaning twig of the *Salvadora persica *tree) instead of toothbrush and fluoridated toothpaste. Our results show that there is a need for preventive programs to improve oral hygiene levels, bearing in mind that the ultimate goal is to prevent more severe periodontal disease prevalence, which is complex to treat.

The mean DMFT 8.7 (SD, 5.9) of our study according to WHO criteria can be considered as low in the 35-44-year age group when compared with same age groups with dental caries levels worldwide. In world map of dental caries prevalence published by the WHO [1], a mean DMFT < 5.0 is considered very low, 5.0-8.9 low, 9.0-13.9 moderate, and > 13.9 high. Our results are slightly higher than other African countries such as Niger (mean DMFT = 5.7) and Uganda (mean DMFT = 3.4) [13,14] but lower than Madagascar (mean DMFT = 13.1) [12].

The mean number of missing teeth in the age group 35-44 years was 4.2 (SD, 4.1), which is in agreement with the study carried out in Madagascar (4.8) but much higher than Uganda (0.6) and Niger (0.4). The finding that the filled component of DMFT was only 0.2% in total population gives an indication of how little dental treatment is actually done, which has important implications for service planning and advocacy.

Virtually all community prevention programmes in Sudan target children and adolescents and as a result, decay among adults is more likely to remain untreated. The mean number of untreated decayed teeth among Sudanese adults in our study was about 9 times that among 12-year-old schoolchildren (DT, 0.4) [34], underscoring the importance of initiating caries-prevention programs for adults.

Among adults aged ≥ 65 years, one third of exposed root surfaces had root caries lesions in the United Kingdom [6], which is considerably higher than our results where those aged ≥ 65 years had caries on only 12% of exposed roots. One reason for this may be the increased life expectancy in the UK, which exposes teeth to the cumulative effect of dental disease for longer. The lower level of root caries may also be partly explained by the relatively low prevalence of wearing partial dentures (3%), which has been shown adversely to affect the remaining dentition through greater incidence of caries [35].

Even though the percent exposed roots in our study was lower in younger adults, their exposed roots were more affected by decay. This may be because of differences such as diet with increased sugar consumption. Toothwear was recorded when it had progressed through tooth enamel into the dentine because considerable inter-examiner variability has been reported when trying to record wear confined to tooth enamel. Results similar to those of our study were obtained in the UK survey [6], where two thirds of all adults had some wear into dentine on anterior teeth. Moderate wear (extensive involvement of dentine) occurred in 11% of adults and 1% had severe wear. Tooth wear has been considered a problem for individual patients rather than being community based. Albeit the trend that tooth wear is increasingly recognized as problematic, it is difficult to foresee who will be affected and true prevention is therefore difficult to accomplish. Presently, treatment is aimed at limiting further tooth wear in individuals already affected by this condition.

Considering the multivariate analysis, dental caries seems less prevalent in older than younger age groups, even after controlling for the effects of confounding variables. This might be because of the small numbers of people present in that category or that teeth presenting decay had already been extracted. The observation that southern tribes are more likely to present with decay than other tribes suggests a cultural dimension to the pattern of decay. The revelation that frequent attenders have higher odds of having decay could emanate from the implication that they experience more pain due to decay and are therefore more likely to seek treatment. Frequent attendance in this survey is not the same as regular checkups, because 91% of patients only went to the dentist when they experienced pain. Those who did go for regular dental checkups in this study were less likely to present with decay. The importance of regular checkups has also been highlighted by others [3] who showed that patients who attended only when they had some trouble with their teeth had one less tooth on average, were twice as likely to have active decay, and six times more likely to have unrestorable caries than those who attended for regular checkups. The finding that people who were educated had lower caries rates is similar to observations made by other authors [25]. Recording the number of ≥ 18 SUNT, which is an arbitrary cut off point, was previously used in adult national oral health surveys in the United Kingdom [7] and Ireland [20]. Western tribes were associated with having lower rates of ≥ 18 SUNT possibly because of some cultural differences or increased consumption of sugar. To clarify the cause further investigations would be necessary. Probands who reported occasional dry mouth also had lower rates of ≥ 18 SUNT. Having a dry mouth has been associated with more decay and tooth loss [36], which might explain the decrease in SUNT observed in the present study.

Increasing age and being male were characteristics associated with periodontal pocketing of ≥ 4 mm. This is consistent with findings from other studies such as the US National Health and Nutrition Examination Survey, 1999-2004 in adults aged 20-64 years. Tobacco users were also more likely to present with periodontal pockets, which is in accordance with other studies by [20,37] wherein smokers had a higher prevalence periodontitis, suggesting poorer periodontal health in these individuals.

## Conclusions

Untreated oral disease was highly prevalent in this study, and we report a low level of literacy, low level of income, high caries prevalence, and lifestyle-related risk factors for oral disease. The low level of literacy might present problems in oral health promotion, which necessitates further development work to determine best delivery mode for health promotion

There was a lack of restorative treatment, which could be due to prohibitively high cost, attitudes on the patients' part, or, prevailing attitude of dentists towards caries management. Containment of disease could be done by simple, minimally invasive, and affordable dentistry. Wider access to restorative dental care could be helped by provision of sufficient manpower and continuing education of dentists to ensure that oral health care providers have sufficient skills and depth of understanding of aspects of oral health care.

As advocated by the WHO, prevention programs to reduce lifestyle-related risk factors of non-communicable diseases such as hypertension, diabetes, and cardiovascular diseases as well as cessation of tobacco use could help prevent some of the oral health outcomes they cause.

## Competing interests

The authors declare that they have no competing interests.

## Authors' contributions

NK: principle investigator was actively involved in the planning, conducting, conception, and design of the study, and had the main responsibility for writing the paper. PFA: was involved in the design of the study as well as manuscript writing. NHA: helped in the study and manuscript writing. MEA: performed statistical analyses. KOA: performed data entry and statistical analysis. All authors have approved the final manuscript.

## Pre-publication history

The pre-publication history for this paper can be accessed here:

http://www.biomedcentral.com/1472-6831/12/5/prepub

## Supplementary Material

Additional file 1 **Distribution of participants' characteristics, their association with and likelihood of having decay, ≥ 18 SUNT, tooth wear, and periodontal pockets ≥ 4 mm**. Click here for file
