# Supplementary material for: A survey of oral health in a Sudanese population
Source: BMC Oral Health. 2012 Feb 24;12:5. doi: 10.1186/1472-6831-12-5 (PMC3311612; doi:10.1186/1472-6831-12-5)
Supplement: Additional file 1 — Distribution of participants' characteristics, their association with and likelihood of having decay, ≥ 18 SUNT, tooth wear, and periodontal pockets ≥ 4 mm. [file 1472-6831-12-5-S1.DOC]

## Distribution of participants’ characteristics, their association with and likelihood of having decay, ≥18 SUNT, tooth wear, and periodontal pockets ≥4 mm

| ***Characteristics*** |  | ***Decay*** | | |  | ***less than 18 SUNT*** | | |  | ***Tooth wear*** | | |  | ***Periodontal pockets of*** | | |  |
| --- | --- | --- | --- | --- | --- | --- | --- | --- | --- | --- | --- | --- | --- | --- | --- | --- | --- |
|  |  |  |  |  |  |  |  |  |  |  |  |  |  | ***4mm +*** | | |  |
|  | ***Total N*** | ***N*** | ***%*** | ***P value*** | ***Odds Ratio*** | ***N*** | ***%*** | ***P value*** | ***Odds Ratio*** | ***N*** | ***%*** | ***P value*** | ***Odds Ratio*** | ***N*** | ***%*** | ***P value*** | ***Odd Rratio*** |
| **Age group** |  |  |  | 0.000* |  |  |  | 0.000* |  |  |  | 0.000* |  |  |  | 0.000* |  |
| 16-24 | 413 | 287 | 69.50 |  | 1 | 11 | 2.7 |  |  | 120 | 29.1 |  | 1 | 23 | 5.60 |  | 1 |
| 25-34 | 616 | 517 | 83.90 |  |  | 27 | 4.4 |  |  | 208 | 33.7 |  | 1.81**(0.29) | 49 | 7.95 |  | 1.48*(0.26) |
| 35-44 | 368 | 334 | 90.80 |  |  | 60 | 16 |  |  | 136 | 36.95 |  | 3.50**(0.75) | 36 | 9.80 |  | 2.07**(0.39) |
| 45-54 | 253 | 227 | 89.70 |  |  | 60 | 24 |  |  | 98 | 38.73 |  | 4.14**(1.13) | 28 | 11.10 |  | 2.10**(0.45) |
| 55-64 | 133 | 111 | 83.50 |  |  | 53 | 40 |  |  | 58 | 43.6 |  | 6.24**(2.49) | 21 | 15.80 |  | 3.60**(0.90) |
| 65-74 | 77 | 62 | 80.50 |  |  | 41 | 53 |  |  | 38 | 49.35 |  | 6.63**(3.54) | 15 | 18.00 |  | 2.61**(0.84) |
| 75 | 22 | 16 | 81.80 |  | 0.16** | 11 | 50 |  |  | 10 | 45.45 |  |  | 3 | 13.60 |  | 5.41**(2.63) |
| **Gender** |  |  |  | 0.008* |  |  |  | 0.000* |  |  |  | 0.062 |  |  |  | 0.025* |  |
| Male | 781 | 666 | 85.3 |  | 1 | 78 | 10 |  | 1 | 117 | 15 |  |  | 210 | 26.9 |  | 1 |
| Female | 1107 | 989 | 89.3 |  | 1.40+(0.26) | 186 | 17 |  | 1.72+(0.55) | 202 | 18.2 |  |  | 248 | 22.4 |  | 0.78*(0.09) |
| **Ethnic group** |  |  |  | 0.007* |  |  |  | 0.000* | 1 |  |  | 0.267 |  | 255 | 23.7 | 0.309 |  |
| North&Central | 1077 | 930 | 86.4 |  | 1 | 157 | 15 |  |  | 173 | 16.1 |  |  | 16 | 18.8 |  |  |
| Eastern | 85 | 69 | 81.2 |  |  | 15 | 18 |  | 1.83*(0.43) | 12 | 14.1 |  |  | 158 | 24.8 |  |  |
| Western | 636 | 573 | 90.1 |  |  | 81 | 13 |  | 2.42+(1.17) | 114 | 17.9 |  |  | 25 | 32.1 |  |  |
| Southern | 78 | 74 | 94.9 |  | 3.07*(1.71) | 9 | 12 |  |  | 19 | 24.4 |  |  | 4 | 33.3 |  |  |
| Others | 12 | 9 | 75 |  |  | 2 | 17 |  |  | 1 | 8.3 |  |  |  |  |  |  |
| **Occupation** |  |  |  | 0.009* |  |  |  | 0.000* |  |  |  | 0.012* |  |  |  | 0.367 |  |
| Semiskilled IV & unskilled V | 1136 | 1001 | 88 |  |  | 198 | 17 |  |  | 208 | 18.3 |  | 1 | 267 | 23.5 |  |  |
| Skilled Manual & Non Manual III | 534 | 479 | 89.7 |  |  | 50 | 9.4 |  |  | 77 | 14.4 |  |  | 135 | 25.3 |  |  |
| Intermediate II | 83 | 69 | 83.1 |  |  | 9 | 11 |  |  | 6 | 7.2 |  | 2.19+(0.97) | 26 | 31.3 |  |  |
| Professional I | 133 | 106 | 79.9 |  |  | 7 | 5.3 |  |  | 28 | 21.1 |  |  | 30 | 22.6 |  |  |
| **Income** |  |  |  | 0.002* |  |  |  | 0.000* |  |  |  | 0.055 |  |  |  | 0.143 |  |
| Low income | 1395 | 1235 | 88.5 |  |  | 228 | 16 |  |  | 247 | 17.7 |  |  | 334 | 23.9 |  |  |
| Moderate income | 397 | 347 | 87.4 |  |  | 26 | 6.5 |  |  | 52 | 13.1 |  |  | 107 | 27 |  |  |
| High income | 96 | 73 | 76 |  |  | 10 | 10 |  |  | 20 | 20.8 |  |  | 17 | 17.7 |  |  |
| **Education** |  |  |  | 0.004* |  |  |  | 0.000* |  |  |  | 0.000* |  |  |  | 0.000* |  |
| No formal schooling | 487 | 441 | 90.6 |  | 1 | 127 | 26 |  |  | 51 | 10.5 |  | 1 | 152 | 31.2 |  |  |
| Primary | 374 | 328 | 87.7 |  |  | 61 | 16 |  |  | 64 | 17.1 |  | 0.70+(0.15) | 97 | 25.9 |  |  |
| Secondary | 528 | 470 | 89 |  |  | 47 | 8.9 |  |  | 97 | 18.4 |  |  | 117 | 22.2 |  |  |
| Higher | 499 | 416 | 83.4 |  | 0.59*(0.15) | 29 | 5.8 |  |  | 107 | 21.4 |  |  | 92 | 18.4 |  |  |
| **Dental visits** |  |  |  | 0.003* | 1 | 18 | 4.2 | 0.000* |  | 86 | 20 | 0.115 |  | 95 | 22.1 | 0.37 |  |
| Never | 429 | 357 | 83.2 |  | 1.39+(0.26) | 198 | 17 |  |  | 179 | 15.6 |  |  | 290 | 25.3 |  |  |
| Less frequent than every 2 years | 1144 | 1012 | 88.5 |  |  |  |  |  |  |  |  |  |  |  |  |  |  |
| More frequent than every 2 years |  |  |  |  | 2.14**(0.59) |  |  |  |  |  |  |  |  |  |  |  |  |
|  |  |  |  |  |  | 48 | 15 |  |  | 54 | 17.1 |  |  | 73 | 23.2 |  |  |
|  | 315 | 286 | 90.8 |  |  |  |  |  |  |  |  |  |  |  |  |  |  |
| **Reason for going to dentist** |  |  |  | 0.000* |  |  |  | 0.201 |  |  |  | 0.1 |  |  |  | 0.027* |  |
| Pain | 1720 | 1570 | 91.3 |  | 1 | 246 | 14 |  |  | 283 | 16.5 |  |  | 429 | 24.9 |  |  |
| Check up | 168 | 85 | 50.6 |  | 0.11**(0.02) | 18 | 11 |  |  | 36 | 21.4 |  |  | 29 | 17.3 |  |  |
| **Tobacco Use** |  |  |  | 0.521 |  |  |  | 0.015* |  |  |  | 0.298 |  |  |  | 0.000* |  |
| No | 1560 | 1364 | 87.4 |  |  | 232 | 15 |  |  | 270 | 17.3 |  |  | 366 | 23.5 |  | 1 |
| Yes | 328 | 291 | 88.7 |  |  | 32 | 9.8 |  |  | 49 | 14.9 |  |  | 92 | 28 |  | 1.57*(0.31) |
| **Dental hygiene** |  |  |  |  |  |  |  |  |  |  |  |  |  |  |  |  |  |
| **Tooth brushing** |  |  |  | 0.06 |  |  |  | 0.067 |  |  |  | 0.619 |  |  |  | 0.103 |  |
| No | 37 | 29 | 78.4 |  |  | 10 | 27 |  |  | 7 | 18.9 |  |  | 5 | 13.5 |  |  |
| Yes 1x/day | 538 | 462 | 85.9 |  |  | 75 | 14 |  |  | 97 | 18 |  |  | 144 | 26.8 |  |  |
| Yes2x/day or more | 1309 | 1160 | 88.6 |  |  | 178 | 14 |  |  | 213 | 16.3 |  |  | 308 | 23.5 |  |  |
| **Mouth rinse** |  |  |  | 0.415 |  |  |  | 0.434 |  |  |  | 0.242 |  |  |  | 0.1 |  |
| No | 1682 | 1479 | 87.9 |  |  | 238 | 14 |  |  | 273 | 16.2 |  |  | 398 | 23.7 |  |  |
| Yes 1x/day | 32 | 26 | 81.3 |  |  | 2 | 6.3 |  |  | 5 | 15.5 |  |  | 7 | 21.9 |  |  |
| Yes2x/day or more | 164 | 141 | 86 |  |  | 24 | 15 |  |  | 35 | 21.3 |  |  | 51 | 31.1 |  |  |
| **Clean between teeth** |  |  |  |  |  |  |  |  |  |  |  |  |  |  |  |  |  |
| No | 1827 | 1605 | 87.8 |  | 1 | 261 | 14 |  |  | 302 | 16.5 |  |  | 445 | 24.4 |  |  |
| Yes 1x/day | 22 | 14 | 63.6 |  | 0.25**(0.13) | 0 | 0 |  |  | 7 | 31.8 |  |  | 5 | 22.7 |  |  |
| Yes2x/day or more | 28 | 27 | 96.4 |  |  | 3 | 11 |  |  | 4 | 14.3 |  |  | 6 | 21.4 |  |  |
| **Use of medication** |  |  |  |  |  |  |  |  |  |  |  |  |  |  |  |  |  |
| No | 1621 | 1432 | 88.3 |  | 1 | 194 | 12 |  |  | 292 | 18 |  |  | 372 | 22.9 |  |  |
| Yes | 267 | 223 | 83.5 |  | 0.67(0.15) | 70 | 26 |  |  | 27 | 10.1 |  |  | 86 | 32.2 |  |  |
| **How often mouth feels dry** |  |  |  |  |  |  |  |  |  |  |  |  |  |  |  |  |  |
| Never | 1526 | 1344 | 88.1 |  |  | 194 | 13 |  |  | 278 | 18.2 |  |  | 365 | 23.9 |  |  |
| Occasionally | 295 | 255 | 86.4 |  |  | 57 | 19 |  | 1 | 35 | 11.9 |  | 1 | 72 | 24.4 |  |  |
| Frequently | 51 | 45 | 88.2 |  |  | 11 | 22 |  | 2.18**(0.57) | 5 | 9.8 |  | 1.43+(0.28) | 17 | 33.3 |  |  |
| Always | 15 | 10 | 66.7 |  |  | 2 | 13 |  |  | 1 | 6.7 |  |  | 3 | 20 |  |  |
| **Had surgery** |  |  |  | 0.992 |  |  |  | 0.002* |  |  |  | 0.163 |  |  |  | 0.085 |  |
| No | 1540 | 1350 | 87.7 |  |  | 197 | 13 |  |  | 269 | 17.5 |  |  | 386 | 25.1 |  |  |
| Yes | 348 | 305 | 87.6 |  |  | 67 | 19 |  |  | 50 | 14.4 |  |  | 72 | 20.7 |  |  |
| **Suffer from disease** |  |  |  | 0.21 |  |  |  | 0.000* |  |  |  | 0.000* |  |  |  | 0.121 |  |
| No | 1562 | 1376 | 88.1 |  |  | 184 | 12 |  |  | 289 | 18.5 |  | 1 | 368 | 23.6 |  |  |
| Yes | 326 | 279 | 85.6 |  |  | 80 | 25 |  |  | 30 | 9.2 |  | 1.60*(0.34) | 90 | 27.6 |  |  |
|  |  |  |  |  | R² = 0.28 |  |  |  | R²= 0.22 |  |  |  | R²= 0.18 |  |  |  | R²= 0.26 |
| *p< 0.05 **significant p-value** from Pearson’s Chi Square test | | | | | |  |  |  |  |  |  |  |  |  |  |  |  |
| +p<0.10; *p<0.05; **p<0.01 **significant exponentiated coefficients (OR);** standard errors in parentheses. | | | | | | | | | | | |  |  |  |  |  |  |
| Some spaces are left blank because only characteristics with increased odds of positive/negative outcome are shown. | | | | | | | | | | | |  |  |  |  |  |  |
